# Exploring the sustainability of quality improvement interventions in healthcare organisations: a multiple methods study of the 10-year impact of the ‘Productive Ward: Releasing Time to Care’ programme in English acute hospitals

**DOI:** 10.1136/bmjqs-2019-009457

**Published:** 2019-07-29

**Authors:** Glenn Robert, Sophie Sarre, Jill Maben, Peter Griffiths, Rosemary Chable

**Affiliations:** 1 Florence Nightingale Faculty of Nursing, Midwifery & Palliative Care, King's College London, London, UK; 2 School of Health Sciences, Faculty of Health and Medical Sciences, University of Surrey, Guildford, UK; 3 Faculty of Health Sciences, University of Southampton, Southampton, UK; 4 Training, Development & Workforce, University Hospital Southampton NHS Foundation Trust, Southampton, UK

**Keywords:** continuous quality improvement, healthcare quality improvement, implementation science, qualitative research, quality improvement methodologies

## Abstract

**Background:**

The ‘Productive Ward: Releasing Time to Care’ programme is a quality improvement (QI) intervention introduced in English acute hospitals a decade ago to: (1) Increase time nurses spend in direct patient care. (2) Improve safety and reliability of care. (3) Improve experience for staff and patients. (4) Make changes to physical environments to improve efficiency.

**Objective:**

To explore how timing of adoption, local implementation strategies and processes of assimilation into day-to-day practice relate to one another and shape any sustained impact and wider legacies of a large-scale QI intervention.

**Design:**

Multiple methods within six hospitals including 88 interviews (with Productive Ward leads, ward staff, Patient and Public Involvement representatives and senior managers), 10 ward manager questionnaires and structured observations on 12 randomly selected wards.

**Results:**

Resource constraints and a managerial desire for standardisation meant that, over time, there was a shift away from the original vision of empowering ward staff to take ownership of Productive Ward towards a range of implementation ‘short cuts’. Nonetheless, material legacies (eg, displaying metrics data; storage systems) have remained in place for up to a decade after initial implementation as have some specific practices (eg, protected mealtimes). Variations in timing of adoption, local implementation strategies and contextual changes influenced assimilation into routine practice and subsequent legacies. Productive Ward has informed wider organisational QI strategies that remain in place today and developed lasting QI capabilities among those meaningfully involved in its implementation.

**Conclusions:**

As an ongoing QI approach Productive Ward has not been sustained but has informed contemporary organisational QI practices and strategies. Judgements about the long-term sustainability of QI interventions should consider the evolutionary and adaptive nature of change processes.

## Background

There is a need for greater insight into the assimilation of quality improvement (QI) interventions into day-to-day healthcare practice and their sustained impact.^1 2^ With rare exceptions, there are few studies of the sustainability of such change interventions in healthcare organisations.^3–5^

The ‘Productive Ward: Releasing Time to Care’ programme is a QI intervention which aims to give ward staff the tools, skills and time needed to implement local improvements in order to: (1) Increase time nurses spend in direct patient care. (2) Improve safety and reliability of care. (3) Improve experience for staff and patients. (4) Make changes to physical environments to improve efficiency. The rationale for Productive Ward was strongly marketed as empowering front-line staff, especially nurses, to ‘take back control’ of their wards and—through efficiency savings—‘release time to care’.

The NHS Institute for Innovation and Improvement in England developed Productive Ward in 2005/2006 for application in hospitals and wards, and was first implemented in 2007.^6 7^ Productive Ward is a self-directed QI toolkit consisting of three foundational modules (‘Well Organised Ward’, ‘Knowing How We are Doing’ and ‘Patient Status At a Glance’) and eight modules dealing with specific ward processes (eg, shift handovers, meals and medicines rounds).^8^ The modules were supplemented by guides for ward, project and executive leaders to aid implementation. Guidance included encouragement to think about sustainability from an early stage,^8^ although a specific ‘sustainability tool’ was not developed until after the initial launch.

As a large-scale QI intervention Productive Ward has three distinctive features. First, it underwent a systematic and extensive design and development process prior to widespread adoption.^7^ It was designed through collaboration with industry partners, drew on social movement theory, and was developed incrementally through piloting and refining modules in collaboration with hospitals.^7^ Second, Productive Ward was adopted very rapidly and widely after being formally launched in January 2008. In May 2008, the government invested £50 million to support the implementation of Productive Ward in all acute hospitals in England.^8^ By May 2012 it was reported that 70% of all acute wards in the UK were implementing Productive Ward.^9^ Third, Productive Ward is remarkable with respect to the scale of the claims made regarding its (potential and achieved) impact. This included a report predicting that a £270 million benefit would result from implementing Productive Ward across acute hospitals in England by March 2014.^10^ However, a decade after the initial development of Productive Ward—and despite its widespread adoption and ongoing use in several other countries—there remains little robust evidence of its impact on acute wards in England. Literature reviews either focus on implementation strategies^11^ or highlight a lack of clarity between activities, perceived impact and measurable outcomes.^12^

Our overall research aim is to explore the impacts and wider legacies of the Productive Ward since its introduction over a decade ago. Responding to a call for more ‘theoretically informative improvement research’^13^ we seek to add to knowledge relating to the implementation and assimilation of QI interventions into routine day-to-day practice, and their sustainability in terms of maintenance of benefits, continuation of activities and continued capacity manifesting at individual, ward and/or hospital level.^14^

## Methods

We conducted a multiple methods study in six case studies comprising purposively sampled acute hospitals. We used multiple sources of information to arrive at ‘best guess’ dates for all adopting English acute hospitals to sample hospitals estimated to have adopted Productive Ward in different years, with the aim of attaining maximum variation. We also sampled for diversity with respect to region, type and size (number of wards) of hospital. Data collection during the period March 2017 to February 2018 comprised a total of: 88 semistructured interviews (with Productive Ward leads, ward staff, Patient and Public Involvement representatives and senior managers); structured observations to note material legacies and observable processes on 12 randomly selected wards (two in each hospital) using a template based on the goals of Productive Ward; and 10 ward manager questionnaires on processes not observable without intrusion, also based on Productive Ward goals. Eligible wards were those that had implemented the three Productive Ward foundation modules and at least one process module. To analyse the interview data we used the Framework method.^15^ Initial themes were developed from: the theoretical literature (see below); the topic guide (itself reflecting theoretical and empirical literature); familiarity with interviews; and inductive coding of four transcripts.

We retrospectively analysed the adoption, implementation, assimilation and legacies of Productive Ward in each of our case studies. In doing so we purposefully distinguished between implementation (formal strategies to promote the integration of interventions into existing practices) and assimilation (the informal process by which, over time, new ideas become part of routine ways of doing things). We categorised the strategies used to implement Productive Ward in each hospital using a published framework which provided a comprehensive categorised list of published implementation strategies^16^ and compared the execution of these strategies against Productive Ward guidance to assess fidelity. We then drew on potential ‘compatibility gaps’ between the assumptions underlying the design and implementation of managerial innovations and the characteristics of adopting organisations^3 17 18^ and the definitions in table 1—as adapted from Kislov^3^—to characterise the interplay between the hospitals and the Productive Ward over time.

**Table 1:** Potential compatibility gaps

Transformation	The adopting hospital modifies its functioning to fit the assumptions behind Productive Ward and the actual use of Productive Ward does not significantly differ from its intended use.
Customisation	Involves both adapting Productive Ward *and* adjusting ward processes.
Loose coupling	Productive Ward is adopted only superficially, in a ritualistic way with the functioning of the hospital remaining largely unaffected.
Co-optation	Productive Ward becomes captured and distorted to serve the interests of the most powerful stakeholders.

Descriptive case studies were written based on the overall framework. Documentary, observational and ward manager questionnaire data were included at this stage. During analysis, within cases we looked for concordances and differences between interviewees’ accounts and between data sets and treated these as data. For example, interviews with lower grades of staff were sometimes at odds with programme leaders’ accounts of their involvement; or observations of wards sometimes belied interview accounts of legacy. We conducted a cross-case analysis of our six case studies, linking legacies to the processes of adoption, implementation and assimilation.

## Results

Below we work backwards by first setting out the legacies of the Productive Ward as we observed them in the six hospitals at the time of our fieldwork (up to 10 years postadoption). We then explain the variation in these legacies by considering the ways in which the Productive Ward was assimilated into routine practice, and how different implementation strategies and timing of adoption may have shaped these long-term organisational processes.

### Legacies of the Productive Ward

#### Material legacies

In each of the six hospitals we observed material legacies of the Productive Ward which remained up to 10 years after initial implementation. Notwithstanding the differing forms of assimilation of the programme in the six hospitals—and how these evolved over time (see below)—we noted that displays of ward metrics data, use of ‘safety crosses’ (to show daily patient harm incidents per month) and storage systems were commonplace on the 12 wards we studied. While maintaining these outward signs of Productive Ward, in day-to-day practice legacies had sometimes become disconnected from their original purpose. For instance, safety crosses were not always clear, up to date or on public display and ‘Knowing How We are Doing’ boards could include data that were difficult to interpret, often out of date and seemingly rarely discussed by ward teams.

#### Process legacies

Our structured observations found that 7 of 18 potential process legacies arising from the modules were still present on all 12 wards (for example, the menu process being conducted outside of mealtimes and the presence of a system for flagging patients who had missed observations). In contrast, three of these potential legacies were not evident on any of the 12 wards; none displayed a ‘ward vision’, written standard operating procedures or an up-to-date ‘Visit Pyramid’ (a graphic display indicating predetermined visits by executive and senior staff which are focused on Productive Ward progress). Ward manager responses to our questionnaires suggested several other standardised processes were commonly still in place (for example, equipment being in the right place and ready to use). The extent to which these processes solely originated with—and have been maintained by—Productive Ward was sometimes unclear. The Productive Ward resources (ie, the guide booklets and toolkit itself) are now rarely used. Nonetheless, staff who had been meaningfully involved in the initial implementation of Productive Ward (mostly Productive Ward leads, ward managers and their deputies) identified wider legacies, such as using Plan-Do-Study-Act improvement cycles, having a ‘lean’ mindset regarding cutting out waste and improving flow, and giving ward staff a greater voice in QI.

#### QI capabilities

Over time the impact of Productive Ward was not confined to individuals or wards; it also shaped QI capabilities within hospitals. By the end of our study whole hospital QI programmes which were at least partially based on Productive Ward were still operational in Hospitals A and C. Hospital A operated a shared governance system to capture staff suggestions for QI while Hospital C was still implementing and developing it’s ‘Trust Quality Bundle’ which had been adapted from Productive Ward. Hospital F’s experience with Productive Ward led them to create both a new Head of Nursing for QI role and a central QI team who delivered training. Former leads of Productive Ward were still offering Productive Ward support to wards in Hospital B, and training in Lean and QI to all staff in Hospitals D and F.

### Assimilation

Table 2 applies Kislov *et al’*s^3^ definitions of the potential ‘compatibility gaps’ to the six hospitals and the Productive Ward during three time periods: at the time of our fieldwork (up to 10 years post-adoption), post-implementation and initial implementation.

**Table 2:** Changing forms of assimilation over time (with illustrative examples)

Hospital	Form of assimilation at time of fieldwork (up to 10 years post-adoption)	Post-implementation (typically year 2 onwards)	Form of assimilation during implementation (typically years 0–2)
A	**Customisation** Whole hospital transformation programme inspired by Productive Ward Hospital-wide system of shared governance to capture staff suggestions on QI	Former facilitators re-deployed to other projects Wards felt that Productive Ward no longer a Trust priority Evolution: of Patient Status At a Glance to e-system; Knowing How We are Doing boards re-evaluated; shift to Accountability Handovers; revisiting processes (some wards)	**Transformation** All wards implemented most modules Standardised Efficient storage system throughout hospital; better stock management; designated areas for equipment; extra equipment purchased Knowing How We are Doing and Patient Status At a Glance boards on all wards Greater staff voice in QI and increased familiarity with data
B	**Customisation** Still part of nursing development lead remit; ad hoc support given to wards by original lead Two ward managers continue to have protected time (1 day a month) Other wards continue to revisit processes, though not using Productive Ward tools Hospital-level discussions underway re-improving use of ward-level data and display	End of funding for leads although continued to support Remaining wards implemented after initial 2-year implementation period Storage overhauled post-implementation Impacts on wards sustained for 1 year Became part of remit of nursing development lead Two ward managers given protected time (1 day a month) to implement on their wards	**Transformation** All wards implemented three foundation modules and at least four process modules ‘Direct Care Time’ reportedly increased in most wards by 15%–20% Standardised efficient storage system; better stock management; designated areas for equipment; extra equipment purchased Knowing How We are Doing and Patient Status At a Glance boards on all wards Greater staff voice in QI
C	**‘Loose coupling’ (see text**) ‘Trust Quality Bundle’ still being implemented and developed. But evidence that ward manager-led (rather than teams); modules seldom rerun Poor reach of staff involvement in QI Standardised Knowing How We are Doing boards still being used (but out of date) Productive Ward storage still in place	Developed a QI ‘bundle’ (‘Trust Quality Bundle’), which used Productive Ward as a framework but incorporated relevant elements of other QI programmes Introduction of Datix web-based incident reporting and risk management software in place of Safety Crosses	**Customisation** Limited number of modules implemented ‘Direct Care Time’ reportedly doubled Knowing How We are Doing boards introduced on all wards; data not used Patient Status At a Glance boards, standardised meals processes/protected mealtimes introduced to all wards Storage and stock control improved
D	**‘Loose coupling’ (see text**) Display and use of data embedded; Safety Crosses still used Electronic Patient Status At a Glance, and standardised Knowing How We are Doing boards still in use Influence on ongoing QI work Lean training available to all staff Limited junior staff engagement with QI	Continued for 12 months Shift handover evolved and ‘Trust Way’ equivalent of Knowing How We are Doing was increasingly tailored to ward New board members marked shift to different QI programme. ‘Trust Way’ leads re-deployed; standard Knowing How We are Doing Boards introduced	**Customisation** Hospital developed own QI tool (‘Trust Way’); consisted of adapted versions of the foundation modules and sustainment process Trust Way extended to non-ward areas Standardised shift handover and protected mealtime policies introduced Poor engagement of junior staff Changes made to physical environment
E	**Loose coupling** Non-standard Knowing How We are Doing and Patient Status At a Glance boards in all wards Safety Crosses still used on some wards, but in some cases ritualistically (not clear or regularly updated) Some ward managers continued to use Productive Ward principles and QI skills	Initial implementation period extended for a further 12 months Implementation team then re-deployed Widespread reorganisation of wards in one hospital, along with staff shortages meant wards there stopped implementing No wards reran any modules once the team had been redeployed	**Loose coupling** No strategic patient public Involvement Limited number of modules implemented Poor engagement with junior ward staff Training given to ward managers only Standardised Patient Status At a Glance boards Changes to physical ward environment Some processes standardised
F	**Loose coupling** Well Organised Ward principles still evident Standardised Knowing How We are Doing boards in all wards but in some cases ritualistically (not relevant or not regularly updated) Safety Crosses still used on some wards, but in some cases ritualistically (not clear or regularly updated) Some ward processes still in place Evidence of ward staff involvement in continuous QI	Operational group set up at the end of the implementation period Productive Ward reported as pivotal in Trust’s decision to set up a QI department Physical infrastructure of the new hospital: Increased mileage walked by ward staff and time spent away from direct care; additional equipment bought to compensate for offward storage Patient Status At a Glance was developed into an electronic system Admissions and discharge work was further developed	**Co-optation** Implementation shaped by requirements of new building (standard layout of wards; single rooms so bedside handover required) Only wards due to move to new building included in roll-ut plan Limited number of modules implemented Wards told which modules to implement Standardised Knowing How We are Doing and Patient Status At a Glance boards introduced to all wards Standardised changes made to storage and stocktaking

QI, quality improvement.

In two of the six hospitals (A and B) we would define what had occurred during the initial implementation period as ‘transformation’; the use of the Productive Ward was as intended (table 2). By the end of the decade ‘customization’ was a more accurate description of how the programme had been assimilated into routine organisational practices in these sites. In Hospitals E and F we found that Productive Ward modules and tools were being used superficially, in a ritualistic way (if at all), with the functioning of the hospitals remaining largely unchanged (what Kislov *et al* would refer to as ‘loose-coupling’^3^). Such ‘loose coupling’ had implications for the relatively limited nature and scale of the legacies we observed in Hospitals E and F.

More nuanced were the stories of the remaining two hospitals. Hospitals C and D adapted Productive Ward significantly while retaining some of the tools; Hospital C also retained the ‘look’ of Productive Ward with its trademark graphic of a house built of (modular) ‘bricks’. Hospital C had radically customised Productive Ward following the implementation period and assimilated the programme in this adapted form with other relevant QI programmes to avoid duplication; modules were modified and new modules added over time to meet new priorities and this continued to operate at the time of our fieldwork (although with some evidence that this may have been something of a paper exercise performed by ward managers on the wards we studied). In Hospital D, which merged its nascent Productive Ward programme with ‘Lean-based’ training it had already commissioned, we found organisational-level legacies of Productive Ward (use of data, availability of training, influence of ongoing QI strategies) but little involvement of lower staff bands in Productive Ward-related activities. We found locating Hospitals C and D within Kislov *et al*’s framework^3^ problematic and return to this issue in our discussion.

### Implementing Productive Ward

#### Fidelity to implementation guidance

Implementation of the Productive Ward was supported by detailed guides for ward, project and executive leaders. We found a consistent failure to extend strategic engagement beyond nursing in their steering groups (see table 3). The—typically—2-year, time-limited availability of funding was usually insufficient to enable hospital-wide implementation in the way that was intended by its designers (particularly in larger organisations with high numbers of wards, see table 4).

**Table 3:** Implementation guidance and fidelity in case study hospitals

Guidance from programme developers*		Levels of fidelity by hospital						Examples of medium/low fidelity
		A	B	C	D	E	F	
Strategic alignment	Define clear goals that align with Trust strategy; secure executive support	Insufficient data						
Implementation and governance structures	Set-up: steering group (including chief executive officer, executive leader, medical director, finance director, general managers, nursing managers); implementation team; ward teams (including matron and representatives from all staff groups)	Medium	Medium	Medium	Low	Medium	Medium	No steering group (D) Steering group did not include medical/ finance directors (A, B, C, F) Matrons omitted from ward team implementation (A) Doctors not engaged at ward level (B, C, D, E) Limited reach/scope of ward staff involvement (D)
Project planning	Create project plan including: roll-out sequence; timetable; resources required; activities; outcomes checklist; progress reviews	High	High	High	High	High	High	
Selecting showcase wards	Invite applications and select showcase wards using the NHSI selection template and sustainability model and guide	High	Medium	Low	Medium	Medium	Low	No application process (B, C, D, E, F) Wards chosen based on patient demographics and incidents (high risk) (C) Readiness to start not measured (F)
Implementation scope	Wards to evaluate current practice with respect to all modules	High	Medium	Low	Medium	High	Medium	Only limited number modules implemented on all implementing wards (typically three foundation modules plus one to three more) (B, C, D, F) Roll-out restricted to half the wards for first 2 years (C)
Productive Ward Leader role	Leading Productive Ward Facilitators; tracking progress and quality; strategic learning; stakeholder management; updating executive leader	High	High	High	High	High	High	
Productive Ward Facilitator role, ward support and training	Support and guide wards and ward managers (not to do the tasks, or act without ward managers’ agreement); provide training	High	High	High	High	Medium	Medium	Productive Ward Facilitators did much of the implementation work (E, F)
Productive Ward Facilitator role (other)	Monitor implementation and measurement; work with central services	High	High	n/a	High	High	High	
Communication	Create and use a communication plan—‘who, what, when, how, why’	Insufficient data						

*These columns derive from the NHS Institute for Innovation and Improvement Executive Leader’s Guide and Project Leader’s Guide.

NHSI, NHS Institute for Innovation and Improvement.

**Table 4:** Study sample: characteristics and resourcing

Hospital	Type of acute hospital	Region in England	Implementation period (start date and duration)	Wards implemented/ total wards	External funding/support	Productive Ward dedicated staff
A	Teaching	Midlands and East	2007 4 years	72/72	External funding from the NHSI for 2 years for 4 PWFs NHSI also funded provision of external support from external delivery partner who helped to plan implementation strategy, trained Productive Ward team, and offered face-to-face support and challenge No backfill for ward staff time	A hospital-wide PWL, a project support officer/data manager (these two posts funded by Trust) and 4 PWFs; all full-time on Productive Ward for at least 2 years Two of the 4 PWFs remained in post for a further 2 years (funded by hospital)
B	Specialist	London	2008 2 years	13/13	SHA funding of approximately £250 000 for: ‘accelerated membership support package’ from NHSI (provided support for up to 10 staff, including 4 days training from NHSI for PWL, executive sponsor & eight ward managers from the early cohorts) A PWL for 2 years Backfill for ward managers implementing Productive Ward Contribution to new equipment costs	1 PWL for 2 years working full-time on Productive Ward
C	Large	South	2007 3 years 2 months	38/40	6 months support from Lean Enterprise Academy funded by NHSI External funding from the NHSI for 1 year for PWL and backfill for ward staff Additional 2 PWFs after first year funded by NHSI for 6 months	1 PWL (funded by NHSI) and 3 PWFs (funded by hospital) full-time for 12 months Additional 2 PWFs for 6 months (funded by SHA) 1.7 PWFs (funded by hospital) for 14 months
D	Small	North	2009 2 years	25/25	No external funding Training in Lean from Unipart Expert Practices 1 Unipart facilitator for 1 year.	1 PWL and 2 PWFs (1 from Unipart) working full-time for 1 year (funded by hospital) One full-time PWF for second year (funded by hospital) Ad hoc support from QI team but no ward staff backfill funding
E	Multiservice	South	2008 2 years 7 months	34/34	Three posts (PWL, PWF, administrator) funded by SHA for 18 months); then extended from charitable funds for a further 12 months No funding for ward staff backfill	1 PWL, 1 PWF and 1 Productive Ward administrator working full-time on Productive Ward for 2½ years.
F	Large	South	2011 1 year 9 months	40/47	No external funding No NHSI training or networking events running by this stage	1 PWL and ‘mentor’ with Productive Ward as part of their remit; and 2 PWFs working full-time (all funded by hospital) for 2 years No funding for ward staff backfill

NHSI, NHS Institute for Innovation and Improvement; PWF, Productive Ward Facilitator; PWL, Productive Ward Leader; SHA, Strategic Health Authority.

There were three key areas in which the hospitals differed. First, hospital A was the only hospital to follow guidance on how it selected early ‘showcase wards’ by inviting and assessing applications based on a ‘sustainability guide’; the five remaining hospitals either relied on nomination by senior nurses or selected wards because they had stable staffing, a keen ward manager or were deemed ‘high risk’. Second, the earliest adopting hospitals, which had either helped develop the materials/toolkit or had extensive training from the original designers of Productive Ward (Hospitals A, B and C) (see table 4) had better engagement across staff bands and groups than other hospitals. Third, only two hospitals (A and E) included all modules in their roll-out plan.

There was considerable variation between wards in the same hospital in terms of implementation. Attitudes of ward managers, and the degree to which they involved staff in any meaningful way, was one issue. Another was that implementation efforts become laboured and Productive Ward teams overstretched as they either began to run out of time to implement all the mandatory modules in their targeted number of wards before their funding ceased or progressed to the more challenging wards and/or time-consuming modules.

#### Timing of adoption and local implementation strategies

Beyond fidelity to the guidance, we found variations in the implementation strategies used by the six hospitals. Applying Waltz *et al*’s categorisation revealed that some elements of local approaches to implementation were common, (for example, ‘providing interactive assistance’^16^) but there were also important differences. First, there was variation in terms of dedicated resources. Timing of adoption was significant in this regard (table 4). Hospitals A, B and C (earlier adopting organisations) received government funding towards salaries for dedicated Productive Ward leads, materials, backfill for ward managers (Hospitals B and C) and expert training; they also had access to supportive networks. In contrast, Hospital F adopted Productive Ward after such funding and networks had ceased. There was wide variation in the numbers of wards in the hospital’s roll-out plans (13 in hospital C to 72 in hospital A, table 4) and the number of modules each ward was expected to implement, and therefore the workload for Productive Ward leads. Whether there was a dedicated lead with time to monitor and encourage ongoing efforts shaped staff engagement over time.

Second, hospitals A, B, C, D and E purchased expert training and support from external providers. In most hospitals training to managers of two to three ‘showcase’ wards was provided by Productive Ward leads and facilitators. But in Hospital B—a small Trust—8 of the 10 ward managers received the expert training directly. It is likely that the relatively high proportion of wards receiving expert training in Hospital B was the reason it stood out in that, first, ward managers continued to implement further modules beyond initial support, and second, it had a notable legacy in terms of staff continuing to make changes according to the underlying principles of Productive Ward. Hospitals A and C—2 years into implementation, in parallel with a reduction in funding—changed their strategy, moving from providing intensive, tailored training to small cohorts of wards towards mass training to ward representatives, module by module. Poor reach of training to all levels of staff in Hospitals E and F meant few staff were demonstrating ‘Productive Ward thinking’ at the time of our fieldwork.

A third key difference was how hospitals evaluated implementation and sustainability, and set up systems to measure outcomes. All hospitals were poor at collating ward-level ‘before implementation’ measures and cost data. After implementation began Hospital A employed a data manager who designed a ward-level data dashboard for capturing monthly outcome measures (although these data were not used strategically at the hospital level). The two hospitals that stood out in terms of evaluative strategies were those where the Productive Ward team were integrated within an existing QI (Hospital D) or change management (Hospital F) team. These hospitals were also notable in forming strategic relationships with central services to aid implementation. Hospital D attempted to build in sustainability from the start of their implementation strategy, using a local system of monthly spot checks, audit sheets, required actions and reporting; in Hospital F ward-level monthly reports included identification of risks to sustainability. Despite this, significant events in each of the two hospitals (changes in the senior executive team in Hospital D and a move to a new hospital location in Hospital F) meant that in both cases Productive Ward—as an active programme—effectively ceased shortly after the implementation period.

The fourth difference in implementation approach was the involvement of patients. While patients were represented on the Productive Ward steering group in Hospitals A, C and F, there was minimal involvement of inpatients on wards in all hospitals. Hospital A, which had a relatively strong history of patient involvement, stood out in how it harnessed patient/public input.

## Discussion

In seeking to address calls to examine both the ‘extent, nature (and) impact of adaptations to … programmes once implemented’^19^ and to ‘yield new theoretical insights applicable to a broader range of settings’,^13^ we have explored (although retrospectively) how adoption, implementation and assimilation processes shaped variations in the legacies of a QI programme in six hospitals over a 10-year period. Our findings emphasise how sustainability is best considered as an evolutionary and adaptive process of change, rather than a stable state.^14^

For the most part, Productive Ward was designed to address the needs of nurses working on wards. The components we typically observed as having been sustained (Well Organised Ward and Patient Status At a Glance boards) were those used—and found useful—by a majority of nursing staff. Productive Ward did also develop QI capacity among those staff meaningfully involved in its implementation. We also found evidence of how the programme helped shape contemporary hospital-wide QI strategies. However, we found several examples of ‘improvements’ being present on wards but only applied in a superficial and ritualistic way (particularly Hospitals E and F). For example, while Productive Ward was undoubtedly part of an emerging movement to make metrics visible and public—through Knowing How We are Doing boards and Safety Crosses—to avoid stasis hospitals need to regularly retrain staff in their use and take advantage of technological developments if intended wider benefits were to be realised on an ongoing basis.

When considering our empirical findings in the light of earlier accounts^3 17–19^ we would propose three refinements to existing theories. First, we agree with Addicott *et al* 18 that there can be ‘temporal flow’ between the compatibility gap typology originally developed by Lozeau *et al*; as Kislov *et al* later noted rather than ‘distinct independent categories’ the four types can indeed ‘represent stages of the same process’.^3^ We would argue that there is now a similar need in recent categorisations of implementation strategies^16 20^ to better recognise an emergent, temporal element and how these interact with assimilation processes to shape legacies of large-scale QI programmes. To illustrate, over time we noted—both within and between our six hospitals—a shift away from a longer-term vision of empowering ward teams to take ownership of the programme (by implementing the modules themselves—‘transformation’) towards a narrower, solely efficiency-based view of the goals of Productive Ward (to be achieved through top-down standardisation and performance measures—‘loose-coupling’, ‘co-optation’); this may explain the limited long-term legacies we observed in the later adopting hospitals who—with fewer resources—appeared to begin from this narrower view. Such shifts and decisions manifested themselves in a range of implementation ‘short cuts’ motivated by time constraints and a managerial desire for standardisation. These short cuts seemed to occur where there were conflicting rationales for the programme among different stakeholders; for example, where a managerial efficiency agenda took precedence over ‘releasing time to care’ on wards. There was often tension between wards’ desire to find their own solutions to their problems, and hospital managers’ desire for standard practices and infrastructure that made it easier for staff to work across a hospital and which could deliver economies of scale (for example, when purchasing equipment). One such ‘short cut’—in hospitals where Productive Ward was relatively under-resourced—saw ward teams excluded from implementation of the programme. For example, in Hospital F facilitators—rather than ward staff—did much of the work including imposing ‘solutions’. Where—as intended—whole ward teams had been trained in the principles underlying Productive Ward (Hospital B and initially in Hospital A) we found that staff continued to apply these principles to their QI work even as organisational contexts changed over time. This finding has parallels with that of Jones *et al* who identified ‘collective change agency’ as one way of closing a compatibility gap between a QI intervention and adopting hospital.^13 19^ More positive legacies were apparent at the time of our fieldwork in those hospitals (A, B, C and D) which enacted (and importantly maintained) higher fidelity to empowering ward-based teams as a fundamental aspect of the recommended implementation approach.

Second, Lozeau *et al* have previously raised a possible contradiction between a rhetoric of empowerment (as in the Productive Ward) and command-and-control procedures for auditing performance data.^17^ Addicott *et al* highlighted empirically how ‘top down’ distortions through ‘performance management systems set up by the remote centre’ limited the intended impact of managed clinical networks for cancer.^18^ In contrast, local Productive Ward leads and managers were important actors who developed their own ward-level monthly implementation reports, as well as local systems of regular audits and monitoring. Rather than distorting the Productive Ward, such locally developed audits—in the absence of remote ‘top down’ guidance or performance measures—did appear to support sustainability. By contrast, it seemed that when ward staff became aware that no one locally was paying attention, work on the programme quickly ceased. This observation contributes to Kislov *et al’*s call for better understanding of how local leaders can balance sensible adaptation of QI interventions with the risk of distortion of core elements of a programme such as Productive Ward.^3^

Third, Jones *et al* have previously suggested that ‘loose-coupling’ between a QI intervention and an adopting hospital can occur when implementation stalls because of simultaneous improvement initiatives overburdening staff.^19^ While this was certainly true in some of the hospitals we studied, the resilience of the QI strategy which was informed by Productive Ward in Hospital C lay in how it absorbed several overlapping initiatives which might otherwise have been competing against each other for limited attention and resources. To a lesser extent we saw similar patterns in Hospitals A and D. Our findings suggest a need to develop and validate a more nuanced classification of assimilation processes than that previously forwarded.^3 17–19^ Intelligent ‘customization’ of the programme at an early stage (Hospitals C and D) gave way to more evolutionary forms of ‘loose-coupling’ where hospitals took account of the assumptions behind Productive Ward to make a wider, positive impact, even though the programme was no longer being used as intended. These were markedly different forms of assimilation to those observed in Hospitals E and F.

Table 5 summarises the implications arising from of our findings for the design and delivery of future large-scale QI programmes.

**Table 5:** Seven lessons for leaders of large-scale quality improvement programmes to consider when reflecting on the story of ‘Productive Ward: Releasing Time to Care’

Think beyond the immediate team	Although many nurses identified with Productive Ward, other staff groups were seldom engaged which undermined ongoing improvements to multidisciplinary processes. In focusing exclusively on ward and nursing processes, the original framing and format of the programme would not meet current demands for multidisciplinary teamwork and system transformation. Involving wider ward teams from the start could have helped mitigate risks to sustainability posed by staff turnover.
Ensure adequate resourcing for task completion and reflection	Funding was needed both to release staff from ward duties (so they could carry out Productive Ward activities) and to enable them to reflect on experiential learning in relation to the underlying principles of the programme (so they could then go on to apply them in changing contexts). The typical 2-year funding period was, in most cases, insufficient to enable implementation of all the modules as intended. A dedicated member of staff was needed to coordinate activities and ongoing training within organisations, as well as demonstrating organisational commitment to the programme. This role was key to realising sustained impacts.
Focus on quality not quantity	Take time to implement foundational modules fully and share lessons learnt and outcomes. As implementation progressed programme leads tended to run out of time and energy, and in later wards ‘solutions’ were imposed by programme leads and/or ward managers without giving other staff opportunities to identify solutions. This led to a sense of ‘being done to’ (rather than shared ownership). Focus on outcomes achieved or lessons learnt, rather than simply recording progress through a staged programme.
Less may be more	Productive Ward would have benefited from being more focused. The modules that were most frequently implemented were: the ‘Foundation modules’ (Well Organised Ward; Patient Status at a Glance; Knowing How We are Doing); followed by four of the process modules (Medicines; Meals; Patient Observations; and Shift Handovers). The remaining four process modules were rarely implemented in any meaningful way.
Play the long game	Adequate before/after and longitudinal data are required to demonstrate impact. Efficient local systems for enabling measurement of impacts and costs (initial and over time) should be built in from the start. The reputation of the Productive Ward suffered to some degree from overclaiming benefits at a national level without a sufficiently longitudinal or robust evidence base. Build considerations of sustainability and develop related guidance into the initial design and testing of a programme.
Adaptability	QI programmes need to be flexible. A programme such as Productive Ward needs to be able to absorb and adapt to changing organisational or system priorities so that relevant learning and resources can be applied to new priorities, rather than entirely new QI programmes being designed to replace or run alongside existing initiatives. Having guidance and toolkits available online where they can be revised and redirected, rather than hard copies of modules, would better support this.
Involve patients and carers as partners	Involve patients, carers and the public. Although guidance suggested roles for patients, visitors and patient representatives at ward and hospital level, such involvement was generally low to non-existent. Recent interest in—and examples of—how co-production can underpin QI work hold important lessons for meaningful and imaginative ways in which service users can and should be part of designing and evaluating programmes like the Productive Ward.

There are several limitations to our study. First, given the retrospective nature of the fieldwork it was sometimes challenging to trace possible legacies of Productive Ward since one or more other QI interventions had often been implemented concurrently or subsequently. Furthermore, wards and hospitals varied with respect to the number of staff available who had been in post during implementation of the programme. Second, assimilation was a challenging concept to explore retrospectively over such a lengthy time period. Lastly, much of our data come from two randomly selected wards in our case study hospitals. Ward practices, perhaps especially QI work, are strongly influenced by ward managers. A different picture of QI work may have emerged at the time of our data collection had we studied other wards.

## Conclusions

We found that Productive Ward has had a lasting impact on specific ward practices; some material and processual changes have remained in place for up to a decade after initial implementation. In this widest sense five of our six case studies could be described as having seen some form of sustained impact from the Productive Ward over the last decade. However, as an ongoing QI approach—continually used to identify and improve problem areas—Productive Ward has been less successful. The resources available at the point of adoption and the—closely related—issue of how the Productive Ward was then implemented locally, shaped the evolving forms of assimilation into routine practice over time; these and wider contextual changes largely determined the legacies of the programme.
